# Robustness of Circadian Clocks to Daylight Fluctuations: Hints from the Picoeucaryote *Ostreococcus tauri*

**DOI:** 10.1371/journal.pcbi.1000990

**Published:** 2010-11-11

**Authors:** Quentin Thommen, Benjamin Pfeuty, Pierre-Emmanuel Morant, Florence Corellou, François-Yves Bouget, Marc Lefranc

**Affiliations:** 1 Laboratoire de Physique des Lasers, Atomes, et Molécules, UFR de Physique, Université Lille 1, Villeneuve d'Ascq, France; 2 Centre National de la Recherche Scientifique, UMR 8523, Villeneuve d'Ascq Cedex, France; 3 Institut de Recherche Interdisciplinaire, Université Lille 1, Villeneuve d'Ascq, France; 4 Centre National de la Recherche Scientifique, USR 3078, Villeneuve d'Ascq, France; 5 Laboratoire d'Océanographie Microbienne, Observatoire Océanologique, Université Pierre and Marie Curie Paris 06, Banyuls/Mer, France; 6 Laboratoire d'Océanographie Microbienne, Observatoire Océanologique, Centre National de la Recherche Scientifique, Banyuls/Mer, France; Universite Libre de Bruxelles, Belgium

## Abstract

The development of systemic approaches in biology has put emphasis on identifying genetic modules whose behavior can be modeled accurately so as to gain insight into their structure and function. However, most gene circuits in a cell are under control of external signals and thus, quantitative agreement between experimental data and a mathematical model is difficult. Circadian biology has been one notable exception: quantitative models of the internal clock that orchestrates biological processes over the 24-hour diurnal cycle have been constructed for a few organisms, from cyanobacteria to plants and mammals. In most cases, a complex architecture with interlocked feedback loops has been evidenced. Here we present the first modeling results for the circadian clock of the green unicellular alga *Ostreococcus tauri*. Two plant-like clock genes have been shown to play a central role in the *Ostreococcus* clock. We find that their expression time profiles can be accurately reproduced by a minimal model of a two-gene transcriptional feedback loop. Remarkably, best adjustment of data recorded under light/dark alternation is obtained when assuming that the oscillator is not coupled to the diurnal cycle. This suggests that coupling to light is confined to specific time intervals and has no dynamical effect when the oscillator is entrained by the diurnal cycle. This intringuing property may reflect a strategy to minimize the impact of fluctuations in daylight intensity on the core circadian oscillator, a type of perturbation that has been rarely considered when assessing the robustness of circadian clocks.

## Author Summary

Circadian clocks keep time of day in many living organisms, allowing them to anticipate environmental changes induced by day/night alternation. They consist of networks of genes and proteins interacting so as to generate biochemical oscillations with a period close to 24 hours. Circadian clocks synchronize to the day/night cycle through the year principally by sensing ambient light. Depending on the weather, the perceived light intensity can display large fluctuations within the day and from day to day, potentially inducing unwanted resetting of the clock. Furthermore, marine organisms such as microalgae are subjected to dramatic changes in light intensities in the water column due to streams and wind. We showed, using mathematical modelling, that the green unicellular marine alga *Ostreococcus tauri* has evolved a simple but effective strategy to shield the circadian clock from daylight fluctuations by localizing coupling to the light during specific time intervals. In our model, as in experiments, coupling is invisible when the clock is in phase with the day/night cycle but resets the clock when it is out of phase. Such a clock architecture is immune to strong daylight fluctuations.

## Introduction

Real-time monitoring of gene activity now allow us to unravel the complex dynamical behavior of regulatory networks underlying cell functions [1]. However, understanding the collective behavior of even a few molecular actors defies intuition, as it depends not only on the topology of the interaction network but also on strengths and response times of its links [2]. A mathematical description of a regulatory network is thus necessary to qualitatively and quantitatively understand its dynamical behavior, but obtaining it is challenging. State variables and parameters are subject to large fluctuations [3], which create artificial complexity and mask the actual network structure. Genetic modules are usually not isolated but coupled to a larger network, and a given gene can be involved in different modules and pathways [4]. It is thus important to identify gene circuits whose dynamical behavior can be modeled quantitatively, to serve as model circuits.

One strategy for obtaining such circuits has been to construct synthetic networks, which are isolated by design [5]–[7]. As recent experiments have shown, an excellent quantitative agreement can be obtained by incorporating when needed detailed descriptions of various biochemical processes (e.g., multimerization, transport, DNA looping, etc.) [7].

Another strategy is to study natural gene circuits whose function makes them relatively autonomous and stable. The circadian clocks that drive biological processes around the day/night cycle in many living organisms are natural candidates, as these genetic oscillators keep track of the most regular environmental constraint: the alternation of daylight and darkness caused by Earth rotation [8]–[11]. Informed by experiments, circadian clock models have progressively become more complex, evolving from single loops featuring a self-repressed gene [12], [13] to networks of interlocked feedback loops [14]–[17].

Here we report surprisingly good agreement between the mathematical model of a single transcriptional feedback loop and expression profiles of two central clock genes of *Ostreococcus tauri*. This microscopic green alga is the smallest free-living eukaryote known to date and belongs to the Prasinophyceae, one of the most ancient groups of the green lineage. *Ostreococcus* displays a very simple cellular organization, with only one mitochondrion and one chloroplast [18], [19]. Its small genome (12.6 Mbp) sequence revealed a high compaction (85% of coding DNA) and a very low gene redundancy [20] (e.g., most cyclins and CDK are present as a single copy gene [21]). The cell division cycle of *Ostreococcus* is under control of a circadian oscillator, with cell division occurring at the end of the day in light/dark cycles [21]. These daily rhythms in cell division meet the criteria characterizing a circadian clock, as they can be entrained to different photoperiods, persist under constant conditions and respond to light pulses by phase shifts that depend on internal time [21].

Very recently, some light has been shed on the molecular workings of *Ostreococcus* clock by Corellou *et al.* [22]. Since the clock of closely related *Arabidopsis* has been extensively studied, they searched *Ostreococcus* genome for orthologs of higher plant clock genes and found only two, similar to *Arabidopsis* central clock genes *Toc1* and *Cca1* [22]. These two genes display rhythmic expression both under light/dark alternation and in constant light conditions. A functional analysis by overexpression/antisense strategy showed that *Toc1* and *Cca1* are important clock genes in *Ostreococcus*. Overexpression of *Toc1* led to increased levels of CCA1 while overexpression of *Cca1* resulted in lower levels of TOC1. Furthermore CCA1 was shown to bind to a conserved evening element sequence (EE) that is required for the circadian regulated activity of *Toc1* promoter. Whether *Toc1* and *Cca1* work in a negative feedback loop could not be inferred from this study since *Ostreococcus* clock appeared to rely on more than a simple *Toc1*/*Cca1* negative feedback loop.

Interestingly, *Arabidopsis* genes *Toc1* and *Cca1* were the core actors of the first plant clock model, based on a transcriptional loop where TOC1 activates *Cca1* and the similar gene *Lhy*, whose proteins dimerize to repress *Toc1* [23], [24]. However, this model did not reproduce well expression peaks of *Toc1* and *Cca1* in *Arabidopsis* [24] and was extended to adjust experimental data [25]. Current *Arabidopsis* clock models feature several interlocked feedback loops [15], [16]. This led us to investigate whether the transcriptional feedback loop model where *Toc1* activates *Cca1* and is repressed by *Cca1* would be relevant for *Ostreococcus*.

We not only found that this two-gene loop model reproduces perfectly transcript profiles of *Ostreococcus Toc1* and *Cca1* but that excellent adjustment of data recorded under light/dark alternation is obtained when no model parameter depends on light intensity. This counterintuitive finding suggests that the oscillator is not permanently coupled to light across the 24-hour cycle but only during specific time intervals, which is supported by numerical simulations. In this article, we propose that the invisibility of coupling in entrainment conditions reflects a strategy to shield the oscillator from natural fluctuations in daylight intensity.

## Results

### Experimental data and model adjustment

**Figure 1:**
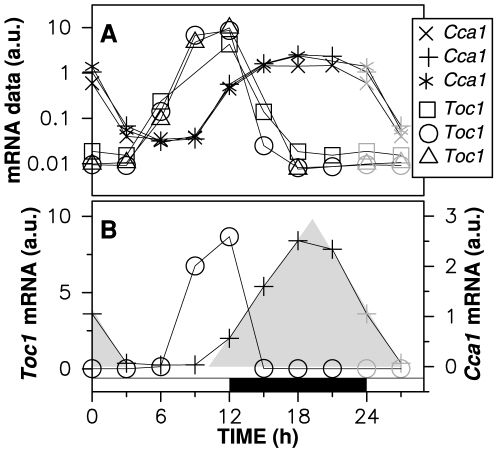
Microarray data recorded under 12∶12 LD alternation. Time zero corresponds to dawn. (A) Experimental data points for the *Cca1* and *Toc1* mRNA time profiles [21] are drawn in logarithmic scale. Data points at zeitgeber time (ZT) 0 and ZT3 have been replicated in gray at ZT24 and ZT27. The target *Toc1* and *Cca1* profiles selected for subsequent analysis are shown with circles and pluses, respectively. These two profiles are also shown in linear scale in (B), where the shaded area illustrates the sawtooth shape of the *Cca1* mRNA profile, which will be used later as evidence of a strongly saturated enzymatic degradation. This area has been obtained by fitting a straight line through *Cca1* data points at ZT12, ZT15 and ZT18 on one hand and at ZT21, ZT0 and ZT3 on the other hand.

To characterize the temporal pattern of *Toc1* and *Cca1* expression in *Ostreococcus*, we used microarray data acquired in triplicate under 12∶12 light/dark cycle, as described in [21] (Fig. 1). One *Toc1* and two *Cca1* mRNA time courses had no aberrant point. Here, we use as target profiles the complete *Toc1* profile and the complete *Cca1* profile whose samples are obtained from the same microarray data as the *Toc1* profile. We checked that the results described in this work are robust to the biological variations observed. Corellou *et al.* have also carried out an extensive work of genetic transformation in *Ostreococcus*, leading to transcriptional and translational fusion lines allowing one to monitor transcriptional activity and protein dynamics in living cells [22]. However, luciferase kinetics in this organism is still not well known and we postpone the analysis of luminescence time series to a future work. Model adjustment has thus been carried out using microarray expression data, which reflect accurately the endogeneous levels of mRNA. Although seeking quantitative agreement with luminescence time series was premature at this stage, predicted protein concentration profiles were compared with data from translational fusion lines as an additional test.

A minimal mathematical model of the two-gene feedback loop comprises four ordinary differential equations (Eq. (2), Methods) with 16 parameters. Since detailed models extending the basic 4-ODE model (2) could only have led to better adjustment, we purposely neglected here effects such as compartmentalisation or delays due to transcription or translation so as to minimize the risk of overfitting and reliably assess the validity of the two-gene loop hypothesis.

Experimental data are recorded under 12∶12 Light/Dark (LD) alternation so that the coupling which synchronizes the clock to the diurnal cycle must be hypothesized. Circadian models usually assume that some parameters depend on light intensity (e.g., a degradation rate is higher in the dark than in the light), and thus take different values at day and night. Parameter space dimension then increases by the number of modulated parameters. Various couplings to light were considered, with 1 to 16 parameters depending on light intensity. We also tested adjustment to model (2) with all parameters constant, which allowed us to quantify the relevance of coupling mechanisms by measuring the difference between best-fitting profiles in the coupled and uncoupled cases.

The free-running period (FRP) of the oscillator in constant day conditions was fixed at 24 hours, which was the mean value observed in experiments [22], but we checked that our main results remain valid for other values of the FRP. In fact, we found that when FRP was freely adjustable, it usually converged to values close to or slightly below 24 hours. Fixing the FRP at exactly 24 hours is interesting in that coupling mechanisms are selected by adjustment only if they improve goodness of fit and not merely to achieve frequency locking.

### A free-running model adjusts experimental data

**Figure 2:**
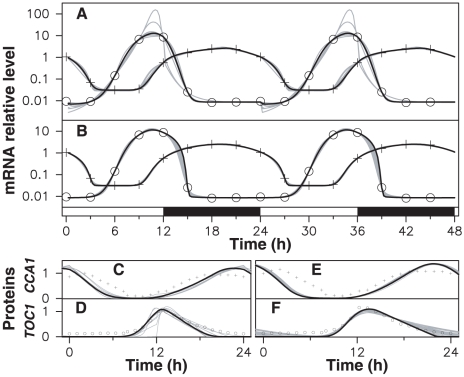
Adjustment of experimental data. The data of Fig. 1(A) are adjusted by model (2) with a FRP of 24 hours. In (A) and (B), crosses (resp. circles) indicate the *Cca1* (resp. *Toc1*) microarray data used as target. Solid lines are best-fitting mRNA time profiles (log scale) obtained with models where (A) all parameters are coupled to light; (B) no parameter is coupled to light; a few solutions near optimum are shown in gray with the best one in black. (C) (resp. (E)) solid lines are CCA1 predicted time profile (linear scale) corresponding to (A) (resp. (B)) with the same color code; crosses correspond to luminescence signals from translational fusion lines. (D) and (F) are the same curves as (C) and (E) for the TOC1 protein.

The first result is that an excellent agreement between numerical and experimental profiles is obtained, with a root mean square (RMS) error of a few percent (Figs. 2(A)–(B)). There is no point in extending model (2) to improve adjustment of microarray data, which are compatible with the hypothesis of a *Toc1*-*Cca1* feedback loop. Moreover, the corresponding protein profiles (not adjusted) correlate well with luminescence signals from CCA1∶Luc and TOC1∶Luc translational fusion lines (Figs. 2(C)–(F)).

But the more surprising is that a non-coupled model, where all parameters are kept constant, adjusts experimental data (Fig. 2(B), RMS error 3.6%) essentially as well as a fully coupled model where all parameters are allowed to vary between day and night (Fig. 2(A), RMS error 3.3%). The corresponding parameter values are given in Table 1. When only one or a few parameters were modulated, goodness of fit significantly degraded compared to the uncoupled and fully coupled cases. This indicates that besides being biologically unrealistic, the model with all parameters modulated fits data merely because of its large parameter space dimension, and cannot be considered seriously. Moreover we simulated the transition from LD alternation to constant light (LL) or constant darkness (DD) conditions for this model and found that it still adjusted experimental data well in LL while displaying strongly damped oscillations in DD (Fig. S1). This confirms that adjustment relies on time profiles being close to free-running oscillator profiles and that adjustment by a fully coupled model is in fact accidental.

**Table 1:** Model parameter values.

Symbol	Description	FC (day)	FC (night)	FR
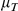	Minimal *Toc1* transcription rate (nM/min)	0.0017	0.0016	0.0065
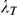	CCA1-dependent *Toc1* transcription rate (nM/min)	0.93	0.29	0.67
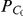	CCA1 level at *Toc1* repression threshold (nM)	1.47	0.00	1.04
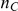	Cooperativity of CCA1	2	2	2
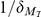	mTOC1 half-life (min)	13.8	22.0	5.08
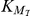	mTOC1 degradation saturation threshold (nM)	8.85	18.3	1.25
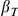	TOC1 translation rate (1/min)	0.013	0.023	0.016
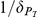	TOC1 half-life (min)	29.9	29.0	3.58
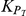	TOC1 degradation saturation threshold (nM)	3.85	9.78	0.76
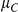	Minimal *Cca1* transcription rate (nM/min)	0.0075	0.017	0.052
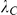	TOC1-dependent *Cca1* transcription rate (nM/min)	0.12	0.047	0.060
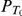	TOC1 level at *Cca1* activation threshold (nM)	100.4	1.49	44.1
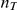	Cooperativity of CCA1	2	2	2
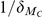	mCCA1 half-life (min)	13.3	52.2	0.82
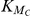	mCCA1 degradation saturation threshold (nM)	0.56	3.76	0.063
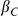	CCA1 translation rate (1/min)	0.056	0.046	0.075
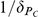	CCA1 half-life (min)	55.5	92.3	54.7
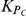	CCA1 degradation saturation threshold (nM)	32.4	36.0	46.0

Parameter values result from adjusting model (2) to experimental data (see Methods) with (i) all parameter values varying between day and night (fully coupled model, FC, Fig. 2(A)) and (ii) all parameter values constant (free-running model, FR, Fig. 2(B)).

On the other hand the uncoupled model is equally unrealistic because it cannot be entrained to the day/night cycle, whereas it is observed experimentally that upon a phase shift of the light/dark cycle, CCA1 and TOC1 expression peaks quickly recover their original timings in the cycle. To verify that adjustment by a free-running oscillator model does not depend on the target profile used, we generated a large number of synthetic profiles whose samples where randomly chosen inside the interval of variation observed in biological triplicates, and adjusted a free-running oscillator model to them. In each case, we found that although RMS error slightly degraded compared our target profile (where mCCA1 and mTOC1 samples for a given time always come from the same microarray), it remained on average near 10%, with visually excellent adjustment (Fig. S2). Last, it should be noted that assuming a FRP of 24 hours allows frequency locking to occur without coupling, but cannot induce by itself best adjustment in this limiting case.

Thus the paradoxical result that data points fall almost perfectly on the temporal profiles of a free-running oscillator is counterintuitive but must nevertheless be viewed as a signature of the clock architecture. As we will see, this in fact does not imply that the oscillator is uncoupled but only that within the class of models considered so far, where parameters of the TOC1–CCA1 loop take day and night values, the uncoupled model is the one approaching experimental data best. Nothing precludes that there are more general coupling schemes that adjust data equally well.

Before unveiling such models, we discuss now whether the simple negative feedback loop described by model (2) is a plausible autonomous gene oscillator. With two transcriptional regulations, it is a simpler circuit than the Repressilator, where three genes repress themselves circularly [5]. It is known that in this topology, oscillations become more stable as the number of genes along the loop increases. The two-gene feedback loop described by (2) could therefore seem to be a less robust oscillator than the Repressilator, and thus a poor model for the core oscillator of a circadian clock.

To address this issue, we checked robustness of adjustment with respect to parameter variations. We found that the experimental profiles can be reproduced in a wide region of parameter space around the optimum, which is quite remarkable given the simplicity of the model (Fig. S3). Moreover, a distinctive feature of the best fitting parameter sets is a strongly saturated degradation, in particular for *Cca1* mRNA, with an extremely low value of 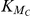 equal to 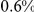 of the maximal *CCa1* mRNA concentration (see Table 1). In this situation, the number of molecules degraded per unit time is essentially constant and does not depend on the concentration except at very small values. This is consistent with the characteristic sawtooth shape of our target profile drawn in linear scale (Fig. 1(B)).

The role of post-translational interactions in gene oscillators and circadian clocks has been recently emphasized (see, e.g., [26], [27]), and in particular saturated degradation has since long been known to favor oscillations [9], [28], [29]. Recently, it has been been shown to act as a delay [30], [31] and to be essential for inducing robust oscillations in simple synthetic oscillators [7], [32], [33] (compare Fig. 1(B) with Fig. 5 of [33]). Thus, strongly saturated degradation is very likely also a key dynamical ingredient of the natural gene oscillator studied here.

### Adjustment by a model with gated coupling

Circadian models are usually coupled to diurnal cycle by changing some parameter values between day and night [12]–[17]. This assumes that all molecular actors involved in light input pathways have been incorporated and that their properties (e.g., degradation rates) react directly to light. Such couplings act over the entire cycle except when light-sensitive actors are present only transiently. For example, models of *Arabidopsis* clock feature an intermediary protein PIF3 that is necessary for induction of CCA1 by light but is shortly degraded after dawn so that CCA1 transcription is only transiently activated [15], [24], [25]. Gating of light input has been observed in several circadian clocks and may be important for maintaining proper timing under different photoperiods [34].

In our case, light/dark alternation has no detectable signature in the dynamics of *Toc1* and *Cca1* mRNA when the clock is phase-locked to the diurnal cycle. This suggests that the actors of the two-gene loop do not sense light directly, and are driven via unknown mediators, which modify their properties inside specific temporal intervals. Since the input pathway can have complex structure and dynamics, possibly featuring separate feedback loops, the windows of active coupling may be located anywhere inside the diurnal cycle and reflect light level at other times of the cycle. Coupling activation should depend both on time of day and on the intrinsic dynamics of the light input pathway, notwithstanding a possible feedback from the circadian core oscillator [35]–[37].

For simplicity, we restrict ourselves to models in which some parameters of the TOC1–CCA1 feedback loop are modified between two times of the day, measured relatively to dawn (ZT0). The start and end times of coupling windows are then model parameters instead of being fixed at light/dark transitions. This assumes that the input pathway tracks diurnal cycle instantaneously, without loss of generality for understanding behavior in entrainment conditions. In this scheme, resetting of the two-gene oscillator can be studied by simply shifting the oscillator phase relatively to the coupling windows. The results so obtained will be sufficient to show that there exist coupling schemes which leave no signature on mRNA profiles, and to study their properties.

What makes our approach original is not the gated coupling to diurnal cycle, which can be found in other models, but the fact that we do not try to model the actors of the input pathway, which can be complex. This is because we focus here on the TOC1–CCA1 feedback loop, which mostly behaves as an autonomous oscillator. Thus we only need to specify the action of the unknown mediators on TOC1 or CCA1, the details of their dynamics being irrelevant.

**Figure 3:**
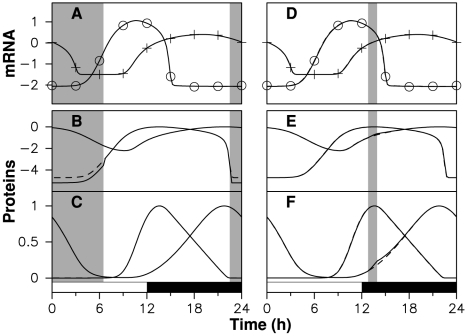
Adjustment by models with gated coupling. Numerical solutions of model (2) without coupling (dashed lines, same parameter values as in Fig 2(B)) and with coupling (solid lines). Gray areas indicate coupling activation. In the left (resp. right) column, TOC1 (resp. CCA1) degradation rate is multiplied by 3 (resp. divided by 2) from ZT22.5 to ZT6.5 (resp. from ZT12.8 to ZT13.95). (A), (D) mRNA time profiles; protein time profiles are shown in (B), (E) logarithmic scale and (C), (F) linear scale.

We systematically scanned the coupling window start and end times, adjusting model for each pair. This revealed that many coupling schemes are compatible with experimental data. For example, TOC1 degradation rate 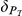 can be modified almost arbitrarily in a large temporal window between ZT22.5 and ZT6.5 without degrading adjustment. This is shown in Figs. 3(A)–(C), where 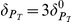 inside this window (here and below, 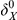 denotes the uncoupled degradation rate of variable 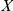). Although the coupling is active for 8 hours, this coupling scheme generates mRNA and protein profiles which are indistinguishable from those of a free-running oscillator. Indeed, modifying TOC1 stability in a window where protein level is low, as is the case for any subinterval of the ZT22.5–ZT6.5 window, does not perturb the oscillator.

We also found a family of time windows of different lengths centered around ZT13.33, inside which the CCA1 degradation rate 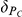 can be decreased without significantly modifying goodness of fit. In Figs. 3(D)–(F), we show the effect of having 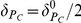 between ZT12.8 and ZT13.95. In this coupling scheme, mRNA profiles are not affected but coupling activation has a noticeable effect on CCA1 level, which rises faster than in the uncoupled case. After the window, however, CCA1 level relaxes in a few hours to the uncoupled profile, losing memory of the perturbation. Near this time of the day, the CCA1 protein level appears to be slaved by the other variables: the perturbation induced by modified degradation does not propagate to the other variables, and when coupling is switched off, the protein level relaxes to its value in the uncoupled solution. Thus, the effect of coupling is not only small but transient. An important consequence, which we will exploit later, is that the two coupling windows shown in Fig. 3 can be combined without modifying adjustment, provided the perturbation induced by one window has vanished when the other window begins.

In these examples, adjustment is sensitive to the timing of these coupling windows: when the start time is modified slightly, the end time must be changed simultaneously so as to recover good adjustment. On the other hand, we found that adjustment error depends little on the coupling strength (measured by the ratio between degradation rates outside and inside the window), especially for short coupling windows.

**Figure 4:**
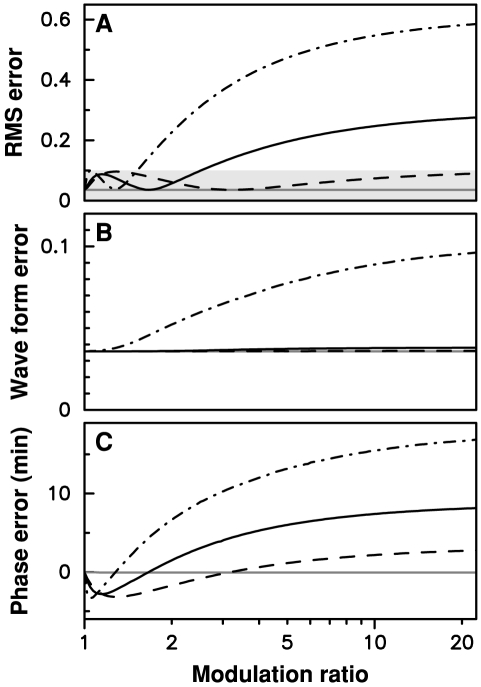
Adjustment error as a function of the coupling amplitude for three coupling windows. (A) In gray, RMS error when 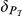 is multiplied by 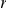 from ZT22.5 to ZT6.5; in black RMS error when 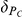 is divided by 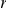 from ZT12.8 to ZT13.95 (solid), from ZT13 to ZT13.65 (dashed), and from ZT12 to ZT15.47 (dash-dotted). The shaded area correspond to adjustment RMS errors below 10%. (B) Waveform error, given by the minimal adjustment error obtained when a variable phase shift is applied to the entrained oscillations; (C) Phase error, defined as the phase shift for which the minimal adjustment error is obtained.

Fig. 4(A) shows how adjustment error varies as a function of coupling strength for the two coupling windows used in Fig. 3 as well as for two other windows inside which the CCA1 protein degradation is reduced, one shorter and the other longer than the window in Fig. 3(B). The window of accelated TOC1 degradation is totally insentitive to modifications of the TOC1 degradation rate, which is due to protein levels being very low in this window. Windows of CCA1 stabilization are all the more insensitive to variations in CCA1 degradation rate as they are shorter. To quantify the sensitivity of a given window we define 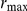 as the largest value of the ratio 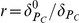 such that adjustment RMS error remains below 10% for any value of 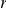 between 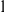 and 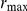. The associated variations in mRNA profiles are visually undetectable and below experimental uncertainties. For the windows ZT12–ZT15.47, ZT12.8–ZT13.95 and ZT13–ZT13.65, of respective durations 3.47, 1.15 and 0.65 hours, we find that the 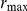 index takes the value 1.5, 2.5 and 260 respectively.

To gain better insight into the effect of a coupling window, we must take into account the fact that the induced variation in the entrained oscillations can be decomposed as a displacement along the limit cycle (resulting in a phase shift) and a displacement transversely to the limit cycle (resulting in a deformation of the limit cycle). To this end, we apply a variable phase shift to the entrained time profile and optimize this phase shift so as to minimize the adjustment error. We define the waveform error as the minimal value of the latter, and the phase error the value of the phase shift for which it is obtained. A small waveform error indicates that we are following the same limit cycle as in the free-running case, possibly with a different phase than is observed experimentally. Waveform and phase errors for the three windows of CCA1 protein stabilization considered in Fig. 4(A) are shown in Figs. 4(B) and 4(C), respectively. It can be seen that only the largest window is associated with a deformation of the limit cycle for large values of 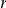, and that it remains modest (RMS error of about 10% for 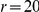). For the two shorter windows, degraded adjustment essentially results from a phase shift of the entrained solution as the modulation index is increased. It can also be seen that the phase error is in fact very small, approximately 7.5 and 2.5 minutes at 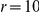 for the two shorter windows. Thus it appears that for short enough windows, the effect of the light coupling mechanism can be entirely captured by studing the phase response induced by the mechanism and that a necessary property of a coupling window is that it induces a zero phase shift of the free-running limit cycle (or a phase shift corresponding to the mismatch between the natural and forcing periods in the general case that we will consider later).

### Systematic characterization of gated coupling mechanisms

Besides the two specific examples shown in Fig. 3, other coupling schemes are compatible with experimental data. In this section, we undergo a systematic approach in order to determine those coupling schemes that do synchronize the free-running model to the day/night cycle, while leaving no signature on mRNA profiles when the phase-locking regime is achieved. To this aim, a preliminary step is to identify coupling schemes which synchronize in the limit of weak forcing using the tools of infinitesimal phase response curve, which can be defined in the framework of perturbation theory in the vicinity of periodic orbits [38]–[40]. Computation of the parametric impulse phase response curve [41] (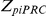) characterizing a light-coupling mechanism corresponding to parameter variation 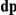 allows one to determine time intervals specified by duration 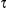 and median position 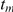 such that when the mechanism is applied in this time interval, it generates a zero phase shift and phase-locking is stable to small perturbations (Text S1). Such intervals satisfy:
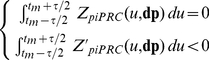
(1)


**Figure 5:**
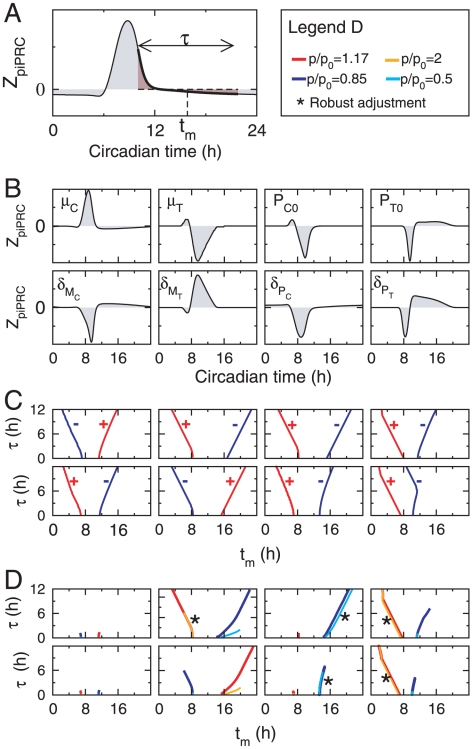
Characterization of coupling schemes. (A) Schematic representation of how window center and duration 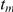 and 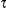, which characterize a coupling with rectangular gating profile, are estimated from the piPRC using Eq. (1). (B) piPRC characterizing the phase change induced by an infinitesimal perturbation of some parameters of the model (transcription an degradation kinetics). (C) Characterization of window center 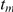 and duration 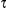 satisfying Eq. (1) for the coupling mechanisms shown in (B), as illustrated in (A). Parameters chosen in (B) are modulated either positively (red) or negatively (blue). (D) Characterization of window center and duration of gated couplings which adjust experimental data with a RMS error below 10% for two different coupling strengths (see box on the right-hand side of the top: 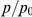 is the ratio between the parameter values within and outside the coupling window).

Figure 5 depicts the properties of various gated couplings in the case where the light-coupling mechanism is assumed to modulate specifically a single transcription-related or degradation-related kinetic parameter. For sufficiently weak positive or negative modulation of those eight parameters, a coupling window of specific width (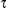) and position (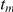) can always be found to satisfy the Eq. 1 (Figs. 5(A)–(C)), thus being compatible with experimental data. However, the adjustment of these weak coupling schemes to data is expected to deteriorate progressively when coupling strength is increased, because (i) the locking phase may change, (ii) the modulation may deviate significantly the trajectory from that of the free-running oscillator or (iii) the entrained solution may loose its stability. Numerical simulations performed at different coupling strengths indicate that only a subset of coupling schemes determined in the limit of weak coupling keep a good adjustement irrespective of the coupling strength. Fig. 5(D) shows window timings such that adjustment error remains below 10% when the kinetic parameter is multiplied or divided by 1.17 or 2. Such a goodness of fit can only be obtained if limit cycle deformation remains small.

As with the examples considered in the previous section, some coupling mechanisms have robust adjustment properties in that a good adjustment is obtained at the two different coupling strengths for the same timings, which coincide with the timings computed in the weak coupling limit. In these cases, adjustment is robust to variations in the coupling strength, which suggests that for these coupling mechanisms, the weak coupling approximation remains valid up to large coupling strengths. For instance, light coupling mechanisms that temporarily increase TOC protein degradation (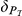) or CCA1 activation threshold (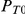) in windows located during the day appear to be robust couplings. Similarly, decreasing CCA1 protein degradation (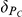) or TOC repression threshold (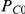) in windows occuring during the night are robust light-coupling mechanisms. Some other mechanisms do not display the same robustness because either the window timings corresponding to good adjustment depend sensitively on coupling strength (e.g., for positive modulation of mTOC1 degradation rate) or because no good adjustment can be found except for very short windows (e.g., modulation of mCCA1 degradation rate). Other robust coupling mechanisms can be identified in Fig. S4, in which the coupling mechanisms not considered in Fig. 5 are characterized.

**Figure 6:**
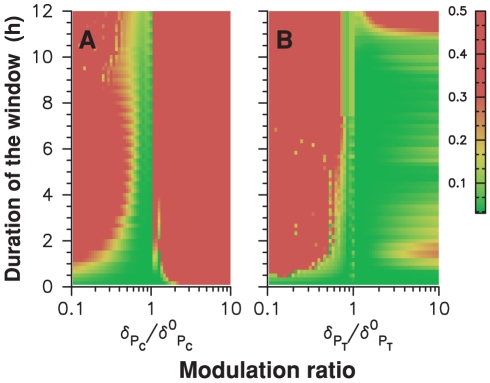
Robustness of adjustment with respect to coupling strength and window duration. Color-coded adjustment RMS error as a function of window duration and modulation ratio (ratio of degradation rates inside and outside the coupling window). (A) Modulation of CCA1 protein degradation rate; (B) Modulation of TOC1 protein degradation rate.

Figure 6 provides a complementary illustration of the robustness of adjustment for models with gated modulation of CCA1 or TOC1 protein degradation rate. In these plots, the window center is kept fixed at the time determined from Eq. (1) and shown in Fig. 5(C) while coupling strength and window duration are freely varied. It can be seen that this timing is compatible with adjustment in a wide range of coupling strengths and window durations.

Our analysis shows that several coupling mechanisms are compatible with the experimental data and that discriminating them requires more experimental data. In particular, monitoring gene expression in transient conditions will probably be crucial since the coupling mechanism leaves apparently no signature in the experimental data in entrainement conditions. For simplicity, we restrict ourselves in the following to models in which half-lives of TOC1 or CCA1 proteins are modified during a specific time interval that is determined in Fig 5(D).

### Resetting

**Figure 7:**
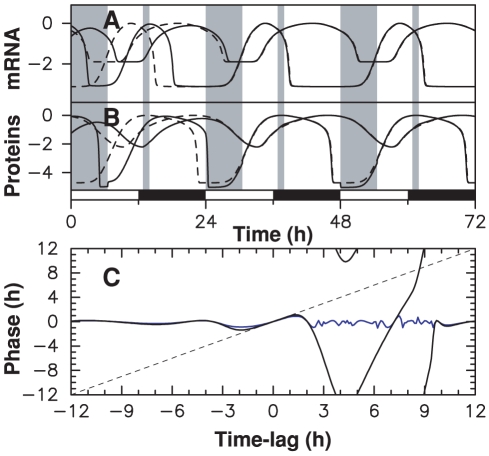
Resetting properties of a model with gated coupling. TOC1 (resp. CCA1) degradation rate is multiplied by 2.1 (resp., by 0.6) from ZT0 to ZT6.5 (resp., from ZT12.8 to ZT13.95). After phase-shifting the day/night cycle by 12 hours, (A) mRNA and (B) protein time profiles (logarithmic scale) of numerical solutions (solid lines) converge rapidly to the nominal profile (dashed lines). (C) Residual phase shift one day (black) and five days (blue) after a phase shift ranging from −12 to 12 hours has been applied.

One may wonder about the purpose of coupling schemes with almost no effect on the oscillator. The key point is that our data have been recorded when the clock was entrained by the diurnal cycle and phase-locked to it. A natural question then is: how do such couplings behave when clock is out of phase and resetting is needed? We found that while the two mechanisms shown in Fig. 3 have poor resetting properties when applied separately (Fig. S5), a combination of both can be very effective. In Fig. 7(A)–(B), we show how the two-gene oscillator recovers from a sudden phase-shift of 12 hours using a two-window coupling scheme. As described above, we assume for simplicity that the two coupling windows remain fixed with respect to the day/night cycle. The 12-hour phase shift is induced by initializing at dawn the oscillator state with the value it takes at dusk in the entrained regime. Figs. 7(A)–(B) show that most of the lag is absorbed in the first 24 hours and the effect of the initial perturbation is hardly detectable after 48 hours.

To design this coupling, we utilized the fact that modifying coupling strengths inside windows hardly affects adjustment. We could therefore choose their values so as to minimize the maximal residual phase shift after three days for all possible initial lags (Fig. 7(C)). Interestingly, we found that the best resetting behavior is obtained when the start time of the window of modified TOC degradation coincides with dawn. Phase locking in this example is globally stable. However, resetting becomes slow when the residual phase shift is under an hour and the residual phase shift is variable (RMS phase error after 5 days is 25 minutes and maximum phase error is 1 hour), and (Fig. 7(C)). This inefficiency results in fact from the limitations of a model where the two parameters are modulated by a rectangular profile with fixed timing. Indeed, we will see later that impressive adjustment and resetting behavior can be simultaneously obtained when parameters are modulated with smooth profiles. Our numerical results thus show that a coupling scheme can at the same time be almost invisible when the oscillator is in phase with its forcing cycle and effective enough to ensure resetting when the oscillator is out of phase. By invisible, we mean that the time profile remains in a close neighborhood of the uncoupled one, so that the only effect of coupling is to fix the phase of the oscillation with respect to the day/night cycle.

### Robustness to daylight fluctuations

Why would it be beneficial for a circadian oscillator to be minimally affected by light/dark alternation in normal operation? A tempting hypothesis is that while daylight is essential for synchronizing the clock, its fluctuations can be detrimental to time keeping and that it is important to shield the oscillator from them. If the entrained temporal profile remains close to that of an uncoupled oscillator at different values of the coupling parameter, then it will be naturally insensitive to fluctuations in this parameter. To gain insight into this fundamental question, we subjected the fully coupled and occasionally coupled clock models to fluctuating daylight.

With the light input pathway unknown, we must allow for the fact that light fluctuations may be strongly attenuated upon reaching the *Toc1*-*Cca1* loop. For example, the light signal could be transmitted through an ultrasensitive signaling cascade with almost constant output above an input threshold close to daylight intensities at dawn. The core oscillator would then be subjected to a driving cycle much closer to a perfect square wave than the intensity profile. We thus considered varying modulation depths for the core oscillator parameters to reflect this possible attenuation.

**Figure 8:**
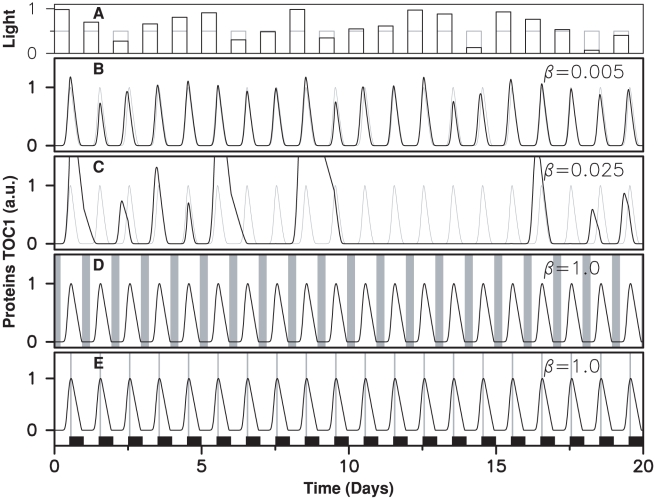
Response of clock models to fluctuating daylight intensity. (A) Light intensity varying randomly from day to day. The time evolution of TOC1 concentration is shown for: (B), (C) the permanently coupled clock model of Fig. 2(A) at two different fluctuation levels, which are quantified by parameter 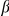 (see Methods); (D) the clock model used in Fig. 3(A)–(C); (E) the clock model used in Fig. 3(D)–(F). When the clock operates nominally, numerical solutions (in black) and experimental time profiles (in gray) superimpose.

Although the two types of model adjust experimental data equally well when subjected to a regular alternation, they have completely different responses to daylight fluctuations. In Fig. 8, we assume that light intensity is constant throughout a given day but varies randomly from day to day. For almost zero modulation, the fully coupled model of Fig. 2(B) maintains relatively regular oscillations of varying amplitude (Fig. 8(B)). When parameter values are modulated by only a few percent, however, this model behaves erratically: oscillations stop for a few days, expression peaks occur a few hours in advance,… (Fig. 8(C)). A circadian clock similarly built would be adversely affected by fluctuations in daylight intensity even with very strong attenuation in the input pathway.

In contrast to this, the two occasionally coupled oscillators of Fig. 3 keep time perfectly even for extreme fluctuations (Figs. 8(D)–(E)) and generate oscillations that are indistinguishable from those of the free-running oscillator which adjusts experimental data recorded under strictly periodic light/dark alternation. Obviously, this extends to models combinining the two windows, such as the one used in Fig. 7. This simple model thus describes a robust clock that is both sensitive to phase shifts in the forcing cycle and insensitive to fluctuations in intensity.

We also studied the effect of fluctuations at shorter time scales. When light intensity was varied randomly each hour, but with the same mean intensity each day, the permanently coupled model was still affected but much less than in Fig. 8 (Fig. S6).

### Influence of free-running period

**Figure 9:**
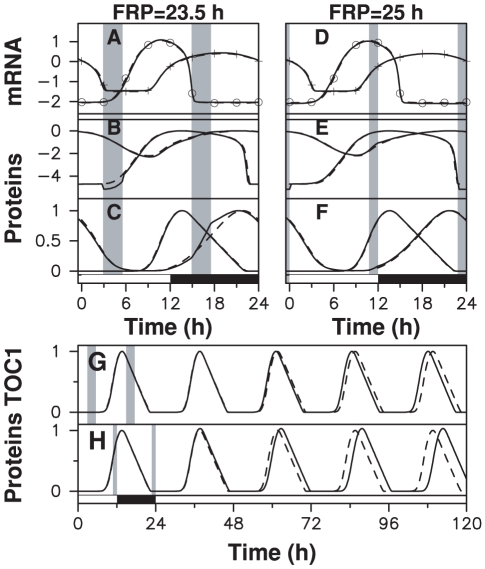
Adjustment by models with gated coupling when FRP is different from 24 hours. Gated coupling can also synchronize free-running clock models with a FRP of 23.5h or 25h without leaving any signature in mRNA profiles. Top left, (A)–(C): numerical solutions of model (2) for a FRP of 23.5h, subjected to coupling windows shown as shaded areas. TOC1 (resp. CCA1) protein degradation rate is multiplied (resp. divided) by three from ZT3 to ZT5.5 (resp. ZT15 to ZT17.5). Top right, (D)–(F): numerical solutions of model (2) for a FRP of 25h, subjected to coupling windows shown as shaded areas. TOC1 (resp. CCA1) protein degradation rate is multiplied (resp. divided) by three from ZT22.75 to ZT24 (resp. ZT11.75 to ZT12). (A), (D) RNA in log scale; crosses (resp. circles) indicate *Cca1* (resp. *Toc1*) microarray data; (B), (E) proteins in log scale; (C), (F) proteins in linear scale. In bottom panel, time evolution of TOC1 protein level (solid lines) during a transition from a 24-hour light/dark cycle to constant light compared to the forced profile (dashed line) for (G) a FRP of 23.5h and (H) a FRP of 25h.

The results described above may seem to rely on the FRP being equal to 24 hours. When the FRP is smaller or larger, coupling is required to achieve frequency locking and pull the oscillation period to 24 hours. To investigate this more general case, we scaled kinetic constants of the free-running model used in Fig. 2(B) to shift the FRP to 25 or 23.5 hours. In both cases (short FRP and long FRP), we could find models with gated coupling that adjust perfectly the experimental data with a period of 24 hours (Fig. 9). These models are very similar to those shown in Fig. 3, the only notable difference being that coupling windows are shifted so that the induced resetting corrects for the period mismatch. Interestingly, the coupling windows for a FRP of 25 hours are located near the light/dark and dark/light transitions. We found that these coupling schemes were also very robust to daylight fluctuations (Fig. S7), indicating that the modulation ratio (equal to 3 for the two windows) is not critical. We also found that without taking adjustment into account, the free running oscillator is entrained by the coupling windows shown in Fig. 9) within a wide range of modulation ratios, from a lower threshold of 1.05 (resp. 1.25) for the FRP equal to 23.5 hours (resp. 25 hours) to an upper threshold of 13 for both FRPs. With a modulation ratio of 3, free-running oscillators with FRPs ranging from 22 to 29 hours could be entrained.

### Gating by smooth profiles

Gating of light input by rectangular profiles does not reflect the fact that the concentration of the mediators modulating the oscillator typically vary in a gradual way. The existence of nested coupling windows such that models with shorter windows can adjust data with larger parameter modulation (see Fig. 4) suggests investigating the action of smooth gating profiles, with maximal parameter modulation near the center of the window. To this end, we considered 24-hour periodic, Gaussian-shaped, modulation profiles defined by: 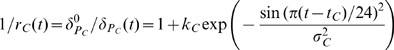 and 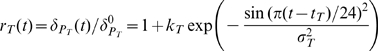, which are parameterized by the times of maximal modulation 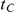, 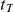, the coupling durations 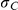, 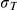 and the modulation depths 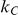 and 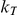. To assess whether good data adjustment and resetting behavior could be obtained simultaneously, these six parameters were chosen so as to minimize the RMS residual phase error 5 days after an initial random phase shift ranging from −12 to 12 hours (see Methods). Note that this naturally forces adjustment to experimental RNA profiles.

**Figure 10:**
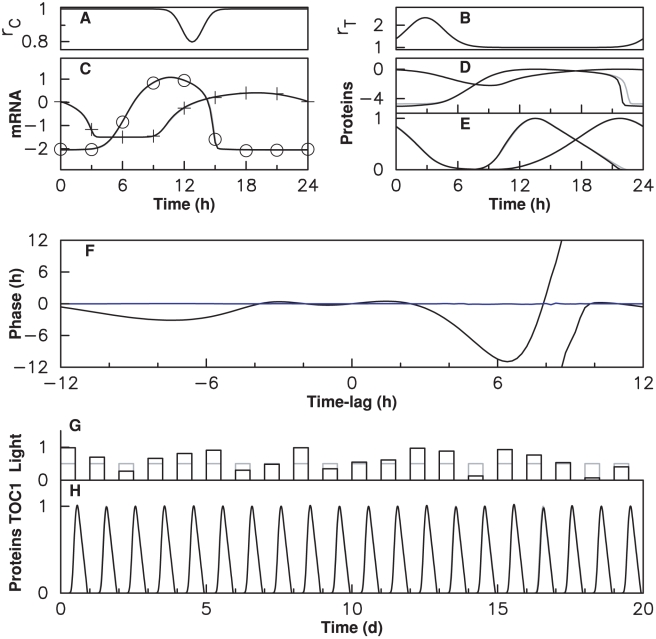
Dynamical behavior of a clock model with gating by Gaussian-shaped modulation profiles. (A) Temporal profile of the CCA1 protein stability modulation coefficient 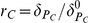 (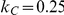, 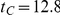, 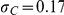) (B) Temporal profile of the TOC1 protein stability modulation coefficient 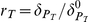 (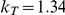, 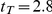 and 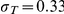). (C), (D) and (E) display numerical solutions of model (2) without coupling (in gray, same parameter values as in Fig 2(B)) and with coupling shown in (A) and (B) (in black). In (C) crosses (resp. circles) indicate the *Cca1* (resp. *Toc1*) microarray data used as target. Protein profiles are shown in (D) (logarithmic scale) and (E) (linear scale). (F): Resetting of the clock after a phase-shift of the day/night cycle. Solid curves display the residual phase shift of the clock after 1 (black) and 5 (blue) day/night cycles as a function of the initial phase-shift. (G) Fluctuating daylight intensity (H) Response of the clock model with smooth coupling profiles to these fluctuations. The protein stability coefficients 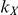 (see Methods) depend on daylight intensity 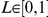 according to 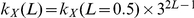.

The behavior of the model using the optimized modulation profiles (Figs. 10(A)–(B)) confirms the findings obtained with rectangular profiles (Fig. 10). The entrained RNA and protein time profiles shadow that of the reference free-running oscillator, with little evidence of the coupling (Figs. 10(C)–(E)). Phase resetting in response to a phase shift is excellent (Fig. 10(F)): RMS (resp. maximum) residual phase shift after 5 days is 2.4 min (resp., 10 min). This is all the more remarkable as the Gaussian shape of the modulation profile is artificial, which shows that the dynamical mechanism exploited here is robust and relatively insensitive to the shape of the modulation profile. Moreover, the oscillator is extremely resistant to daylight fluctuations (Fig. 10(F)). In spite of its simplicity, the two gene-oscillator studied here thus fulfills key requirements for a circadian oscillator when modulated with the right timing.

## Discussion

Our findings illustrate how mathematical modeling can give insight into the architecture of a genetic module. Not only can expression profiles of two *Ostreococcus* clock genes be reproduced accurately by a simple two-gene transcriptional feedback loop model, but furthermore excellent adjustment of mRNA data is provided by a free-running model. This counterintuitive result can be explained if coupling to the diurnal cycle occurs during specific temporal windows, where unidentified mediators interact with the TOC1-CCA1 oscillator in such a way that it experiences negligible forcing when it is in phase with the day/night cycle, and strong resetting when it is out of phase. We could exhibit many coupling schemes compatible with experimental mRNA temporal profiles, differing by the coupling mechanism or by the window timing. This indicates that identification of the actual light input pathway will require additional experimental data. Our analysis strongly supports the conjecture that *Ostreococcus* genes *Cca1* and *Toc1* are the molecular components of an oscillator at the core of *Ostreococcus* clock but does not exclude that other coupled oscillators or feedback loops exist.

Why would a circadian oscillator decouple from the day/night cycle when in phase with it so as to generate quasi-autonomous oscillations? A natural hypothesis is that this protects the clock against daylight fluctuations, which can be important in natural conditions [42]. In a vast majority of numerical simulations and experiments on circadian clocks reported in the literature, the day/night cycle is taken into account through a perfect alternation of constant light intensity and darkness. However, this is somehow idealized, as the primary channel through which clocks get information about Earth rotation, namely daylight, is variable.

In nature, the daylight intensity sensed by an organism depends not only on time of day but also on various factors such as sky cover or, for marine organisms such as *Ostreococcus*, the distance to sea surface and water turbidity, which can affect perceived intensity much more than atmosphere. Therefore, the light intensity reaching a circadian clock can vary several-fold not only from one day to the next but also between different times of the day.

A clock permanently coupled to light is also permanently subjected to its fluctuations. Depending on the coupling scheme, keeping time may become a challenge when fluctuations induce phase resettings and continuously drive the clock away from its desired state. Indeed, we found that a mathematical model with properly timed coupling windows was insensitive to strong light intensity fluctuations while a permanently coupled model became erratic even for very small coupling strengths. For simplicity, we only tested the robustness of a model with modulated TOC1 and CCA1 protein degradation. However, it should be stressed that all other light-coupling mechanisms that were found to be robust with respect to adjustment (see Fig. 5 and Fig. S4) are naturally also robust with respect to daylight fluctuations. Indeed they adjust the experimental data for varying coupling strengths at fixed window timings. This indicates that the limit cycle is insensitive to variations in the coupling strength, which is the key to the robustness to daylight fluctuations. Another interesting result from our numerical simulations is that the most disruptive fluctuations are the variations in intensity from one day to the other, since their time scale matches the oscillator period. Indeed, faster or slower fluctuations are easily filtered out.

These results lead to enquire whether similar designs exist in other circadian clocks. Although the importance of this problem was noted some time ago [42], the robustness of circadian clocks to daylight fluctuations and how this constraint shapes their molecular architecture have been little studied until very recently [43], [44]. The discussion on how genetic oscillators can keep daytime has essentially focused on the most important sources of noise under constant conditions : temperature variations [40], [45], [46] or fluctuations in concentration due to small numbers of molecules [47], [48]. However, an operating clock is naturally subjected to an external forcing cycle, which is yet another source of fluctuations.

We thus conjecture that a circadian clock must be built so as to be insensitive to daylight intensity fluctuations when entrained by the day/night cycle, just as it is insentitive to molecular or temperature fluctuations, and that this can be achieved by keeping the oscillator as close to the free-running limit cycle as possible, scheduling coupling at a time when the oscillator is not responsive. An important consequence of this principle is that it allows us to discriminate between different possible coupling mechanisms for a given model, as our analysis revealed dramatic differences in the ability of different parametric modulations to buffer fluctuations. It also allows us to determine the preferred timing for a given coupling mechanism, which may prove very helpful when trying to identify the molecular actors which mediate the light information to the clock.

When the FRP is close to 24 hours, as in much of our analysis, it is easy to understand why robustness to daylight fluctuations requires that the forced oscillation shadows the free-running solution. Robustness manifests itself in the time profile remaining constant when subjected to random sequences of daylight intensity. This includes strongly fluctuating sequences as well as sequences of constant daylight intensity at different levels. Thus, the oscillator response should be the same at high and low daylight intensities, which implies that the solution must remain close to the free-running one as forcing is increased from zero. Note that this only holds in entrainment conditions, where coupling is not needed. When the clock is out of phase, strong responses to forcing are expected, with resetting being faster as forcing is stronger.

When the natural and external periods are significantly different, the problem may seem more complex as coupling is required to correct the period mismatch. There is a minimal coupling strength under which the oscillator is not frequency-locked and entrainment cannot occur. Nevertheless, we showed that timing the coupling windows properly is as effective for oscillators with FRP of 23.5 and 25 hours as for the 24-hour example we had considered. Again, the forced solution remains close to the free-running limit cycle even if proceeding at a different speed to correct the period mismatch. This also shows that FRP is not a critical parameter for adjustment of the experimental data used here.

A consequence of the small deviation of the limit cycle from the free-running one when coupling strength is varied is that oscillations should vary little upon a transition from LD to LL or DD conditions (see, e.g., Figs. 9(G)–(H)). We searched the litterature for examples of such behavior. Ref. [13] provides a interesting comparison of models for the *Drosophila* and *Neurospora* circadian clocks which is illustrative for our discussion. In this study, the variation in amplitude is much less pronounced for the *Drosophila* model than for the *Neurospora* one (see Fig. 2 of [13]). Concurrently, the sensitivity of the phase of the entrained oscillations to variations in the light-controlled parameter is much smaller for the *Drosophila* model (see Fig. 3 of [13]), which is a necessary condition for robustness to daylight fluctuations. Another interesting comparison involves the one-loop and two-loop models of *Arabidopsis* clock [24], [25]. The one-loop model clearly modifies its behavior upon entering DD conditions from LD (see Fig. 5 of [24]) while the two-loop model preserves its average waveform when transiting from LD to LL, except for the disappearance of the acute response to light at dawn (see Fig. 6 of [25]). Thus, the two-loop model not only reproduces experimental data better but also seems more robust.

The *Drosophila* and *Neurospora* clock models analyzed in [13] also differ in their response to forcing when their FRP is close to 24 hours [49]. A number of circadian models cannot be entrained when their FRP is too close to 24 hours because complex oscillations, period-doubled or chaotic ones, are observed easily for moderate to strong forcing. Indeed, it is expected that near resonance between the forcing and natural periods, the strong response exalts nonlinearities and favors complex behavior. Again, the *Drosophila* clock model appears to be more robust in this respect [49]. We stress that making the coupling invisible in entrainment conditions naturally addresses this issue. Dynamically uncoupling the oscillator from the diurnal cycle in entrainment conditions makes it immune both to fluctuations in daylight intensity and to destabilization in the face of strong forcing.

An important problem is how a clock with occasional coupling can adjust to different photoperiods so as to anticipate daily events all along the year. We can only touch briefly this question here as it requires understanding how the temporal profile of the coupling windows changes with photoperiod and thus a detailed description of the unknown light input pathways and additional feedback loops that control the timing of these windows. The key point is that the phase of the entrained oscillations is controlled by the position of the coupling windows. Thus the role of light input pathways and additional feedback loops, whose internal dynamics will typically be affected by input from photoreceptors and feedback from the TOC1–CCA1 oscillator, is to time the coupling windows as needed for each photoperiod so that the correct oscillation timing is generated [35]–[37]. This question will be addressed in a future work, together with the analysis of the luminescence time series recorded for differents photoperiods.

Our results also bring some insight into the recent observation that a circadian clock may require multiple feedback loops to maintain proper timing of expression peaks in response to noisy light input across the year [43]. We have shown here that a single two-gene loop can display impressive robustness to daylight fluctuations when its parameters are modulated with the right timing. As noted when discussing the response to different photoperiods, this requires the presence of additional feedback loops to generate the biochemical signal needed to drive the core oscillator appropriately, and which we have not yet identified and modeled in *Ostreococcus*. Robustness to fluctuations thus implies a minimal level of complexity.

Finally, robustness to intensity fluctuations may explain why it is important to have a self-sustained oscillator at the core of the clock, as a forced damped oscillator permanently needs forcing to maintain its amplitude, and is thereby vulnerable to amplitude fluctuations. Confining the dynamics near the free-running limit cycle allows to have a pure phase dynamics for the core oscillator, uncoupled from intensity fluctuations. Understanding how to construct it will require taking into account the sensitivity of the free-running oscillator to perturbations across its cycle [50].

A simple organism as *Ostreococcus* can apparently combine mathematical simplicity with the complexity of any cell. The low genomic redundancy of *Ostreococcus* is certainly crucial for allowing accurate mathematical modeling, leading to better insight into the clock workings. *Ostreococcus* therefore stands as a very promising model for circadian biology, but also more generally for systems biology.

## Methods

A minimal mathematical model of the transcriptional loop where *Toc1* activates *Cca1* which represses *Toc1*, consists of the following four differential equations:
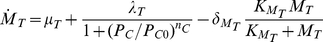
(2a)

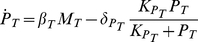
(2b)

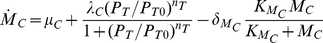
(2c)

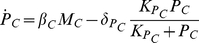
(2d)Eqs (2) describe the time evolution of mRNA concentrations 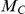 and 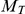 and protein concentrations 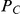 and 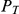 for the *Cca1* and *Toc1* genes, as it results from mRNA synthesis regulated by the other protein, translation and enzymatic degradation. *Toc1* transcription rate varies between 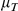 at infinite CCA1 concentration and 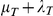 at zero CCA1 concentration according to the usual gene regulation function with threshold 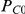 and cooperativity 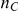. Similarly, *Cca1* transcription rate is 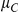 (resp., 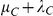) at zero (resp., infinite) TOC1 concentration, with threshold 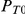 and cooperativity 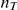. Translation of TOC1 and CCA1 occurs at rates 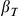 and 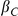, respectively. For each species 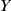, the Michaelis-Menten degradation term is written so that 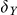 is the low-concentration degradation rate and 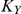 is the saturation threshold.

Model (2) has 16 free continuously varying parameters besides the cooperativities 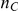 and 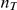 which can be set to the integer values 1 or 2 by the adjustment procedure. mRNA concentrations are determined experimentally only relative to a reference value and protein profiles are not adjusted. Therefore, two solutions of Eqs. (2) that have the same waveforms up to scale factors are equivalent. Therefore, we can eliminate four parameters by scaling Eqs. (2), with only 12 free parameters controlling adjustment when parameters do not vary in time, which optimizes parameter space exploration. Then parameters are rescaled so that the maximum value of protein profiles is 100 nM, the maximum value of *Cca1* mRNA profile is 10 nM, and the *Toc1* and *Cca1* mRNA maximum values are in the same proportion as in microarray data. This makes it easier to compare regulation thresholds and degradation saturation thresholds relative to the maximum values of the four concentrations. When the number of modulated parameters is 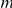, parameter space is 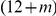-dimensional.

Adjustment was carried out by using a large number of random parameter sets as starting points for an optimization procedure based on a Modified Levenberg–Marquardt algorithm (routine LMDIF of the MINPACK software suite [51]). Goodness of fit for a given parameter set was estimated by the root mean square (RMS) error between experimental and numerical mRNA levels, in logarithmic scale. Numerical integration was performed with the SEULEX algorithm [52]. Adjustment was carried out with 14 (resp. 2) Quad-Core Intel Xeon processors at 2.83 GHz during 72 hours for the 28-dimensional (resp. 12-dimensional) parameter space. Convergence was checked by verifying that the vicinity of the optimum was well sampled. In the uncoupled case, the ODE system is invariant under time translation so that its solutions are defined up to an arbitrary phase. An additional routine was then used to select the best-fitting phase.

To study the effect of daylight fluctuations, parameters were modulated as follows. 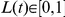 is the randomly varying light intensity, with 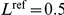 the reference level. We define the reference modulation depth of the 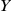 parameter taking value 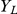 at standard light level and 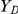 in dark as 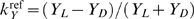. 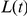 modifies modulation depth according to 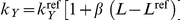, where 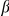 quantifies sensitivity to light variation. The modified modulation depth fixes a new value for the day value, the dark value being unchanged. For models with occasional coupling, we use similar definitions with dark and light parameter values replaced by parameter values respectively outside and inside of the coupling window. The CCA1 stability modulation inside the window starting after dusk depends on the intensity of the previous day.

The parameters of the Gaussian-shaped modulation profiles were determined by optimizing resetting. For all possible variable initial time lag ranging from −12 to 12 hours, the effect of the coupling scheme based on the two profiles modulating TOC1 degradation and CCA1 degradation was characterized as follows. The time lag was applied to the free-running cycle adjusting experimental data. Then, the coupling scheme was applied for one or 5 days. Finally, the coupling was switched off and the residual phase error was measured after two days. The set of six parameters defining modulation profiles were obtained as those which minimize RMS residual phase error across the 24-hour interval.

## Supporting Information

Figure S1 Transition from light/dark alternation(LD) to constant light (LL) and constant darkness (DD) for the fully coupled model. Time evolution of mRNA concentrations for the fully coupled model shown in Fig.∼2(A) for various light protocols: LD alternation (dashed, black), one LD period from ZT0 to ZT24 then constant light (in red) and one LD period from ZT0 to ZT24 then darkness (in blue). *Cca1* and *Toc1* mRNA concentrations are shown in the top and bottom frame, respectively.
(0.02 MB PDF)

Figure S2 Influence of experimental errors on adjustement of a free running oscillator model to data. Alternate target profiles with samples randomly chosen inside the interval of variation observed are generated and adjusted. Each random target corresponds to a slightly different parameter set and to a different adjustment RMS error (A) RMS error distribution; (B) The five target profiles most distant from each other have been selected and are associated with different colors. Crosses (resp. circles) indicate the *Cca1* (resp *Toc1*) mRNA target samples, the solid line is the numerical solution of the adjusting model.
(0.03 MB PDF)

Figure S3 Probability distribution for parameter values in parameter sets with adjustment RMS error below 10%. Parameters are determined as explained in Methods. The percentage of occurrence is evaluated for bins of width 0.2 in log_10_. The probability distributions of parameter values for the model with all parameters modulated are shown in red and blue for the day and night values, respectively. The probability distribution of parameter values for the model with all parameters constant is shown in black.
(0.02 MB PDF)

Figure S4 Characterization of coupling schemes. (A) iPRC characterizing the phase change induced by an infinitesimal perturbation of parameters *λ_X_*, *β_X_* and *K_X_*. (B) Characterization of time position, *t_m_*, and duration *τ* of couplings with a rectangular gating profile satisfying Eq. (1). Parameters are modulated either positively (red) or negatively (blue). (C) Characterization of time position and duration of couplings with a rectangular gating profile adjusting experimental data with a RMS error below $10% for four different levels of coupling strength (blue: *p/p_0_* = 1.17; cyan: *p/p_0_* = 2; red: *p/p_0_* = 0.85; orange: *p/p_0_* = 0.5; *p/p_0_* being the ratio between the parameter values within and outside the coupling window.
(0.03 MB PDF)

Figure S5 Resetting of the clock model of Fig. 4 in response to a phase shift of the day/night cycle. Solid curves display the residual phase shift of the clock after 1 (black) and 5 (blue) day/night cycles as a function of the initial phase shift. (A) TOC1 degradation rate is multiplied by 2.1 between ZT0 and ZT6.5. (B) CCA1 degradation rate is multiplied by 0.6 between ZT12.8 and ZT13.95. (C) Figure 6C is reproduced here for convenience. TOC1 (resp. CCA1) is multiplied by 2.1 (resp. 0.6) between ZT0 and ZT6.5 (resp. ZT12.8 and ZT13.95), which results in uniform convergence to phase-locking. Phase RMS error after 5 day/night cycles is 25∼min while the maximum error is 1∼hour.
(0.03 MB PDF)

Figure S6 Response of the fully coupled and occasionally coupled clock models to fluctuations in daylight intensity occurring on a time scale of one hour. The figure is otherwise similar to Fig 8.
(0.03 MB PDF)

Figure S7 Response of the two occasionally coupled clock models of Fig.∼8 to fluctuations in daylight intensity. (a) Light intensity varying randomly from day to day. The time evolution of TOC1 protein concentration is shown for: (b) the clock model with a FRP of 23.5h; (c) the clock model with a FRP of 25h. The figure is otherwise similar to Fig∼8.
(0.06 MB PDF)

Text S1 Characterization of gated coupling mechanisms in the weak modulation limit.
(0.07 MB PDF)
