# Supplementary figures and images for: Robustness of Circadian Clocks to Daylight Fluctuations: Hints from the Picoeucaryote Ostreococcus tauri
Source: PLoS Comput Biol. 2010 Nov 11;6(11):e1000990. doi: 10.1371/journal.pcbi.1000990 (PMC2978692; doi:10.1371/journal.pcbi.1000990)

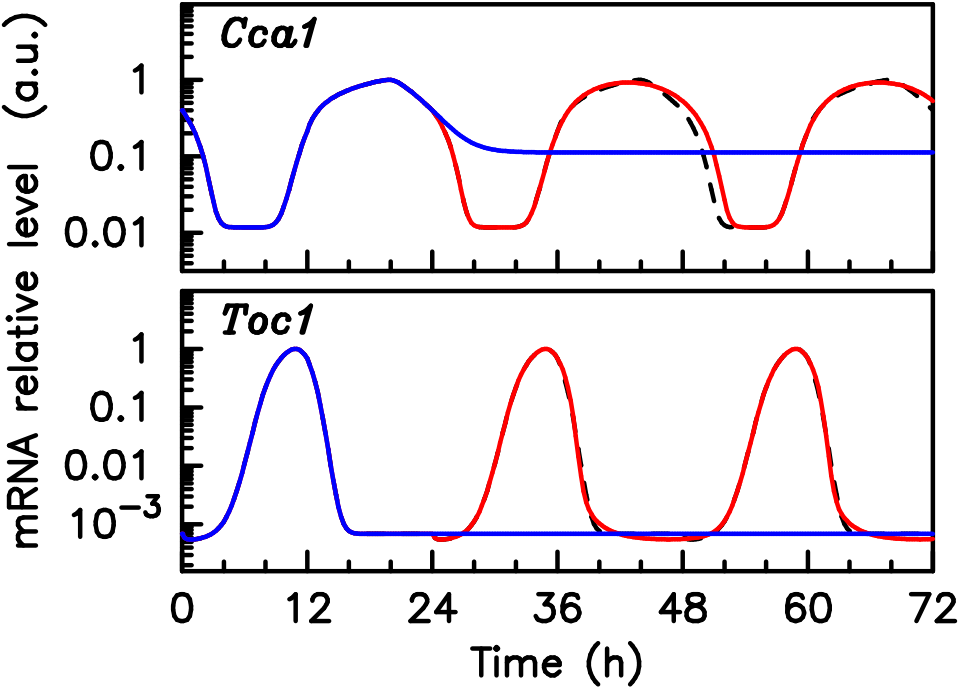

Supplement: Figure S1 — Transition from light/dark alternation(LD) to constant light (LL) and constant darkness (DD) for the fully coupled model. Time evolution of mRNA concentrations for the fully coupled model shown in Fig.∼2(A) for various light protocols: LD alternation (dashed, black), one LD period from ZT0 to ZT24 then constant light (in red) and one LD period from ZT0 to ZT24 then darkness (in blue). Cca1 and Toc1 mRNA concentrations are shown in the top and bottom frame, respectively. (0.02 MB PDF) [file pcbi.1000990.s001.pdf]

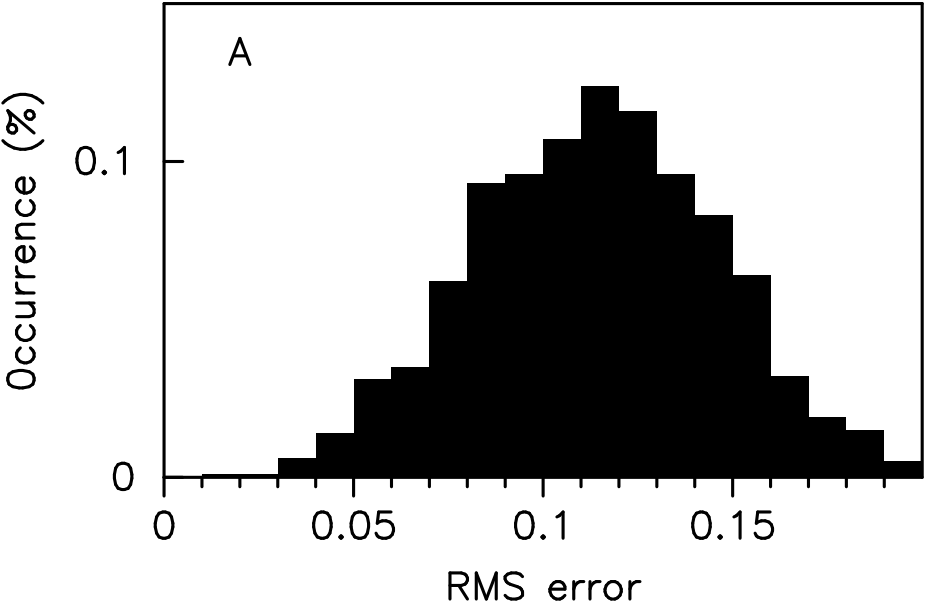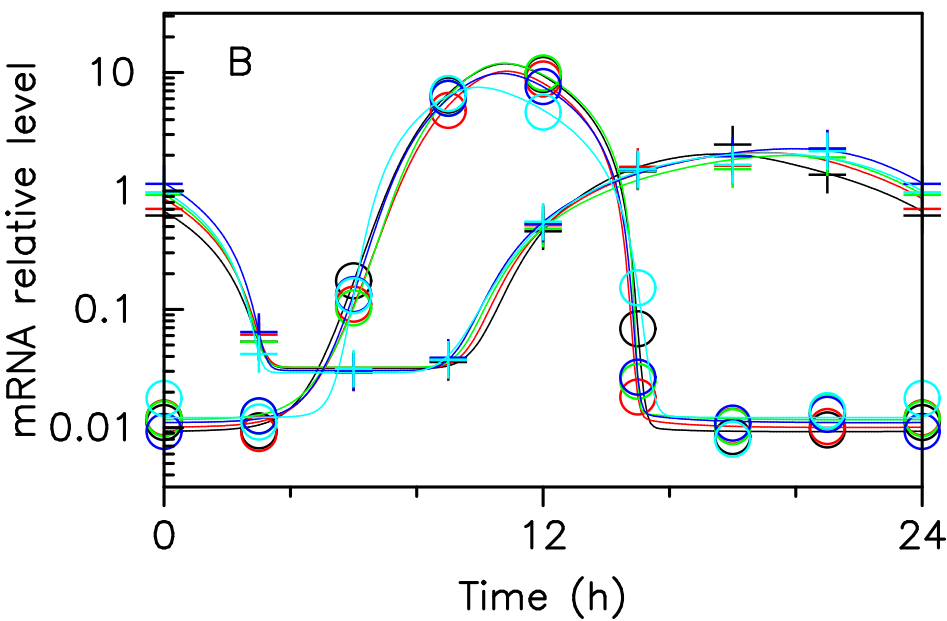

Supplement: Figure S2 — Influence of experimental errors on adjustement of a free running oscillator model to data. Alternate target profiles with samples randomly chosen inside the interval of variation observed are generated and adjusted. Each random target corresponds to a slightly different parameter set and to a different adjustment RMS error (A) RMS error distribution; (B) The five target profiles most distant from each other have been selected and are associated with different colors. Crosses (resp. circles) indicate the Cca1 (resp Toc1) mRNA target samples, the solid line is the numerical solution of the adjusting model. (0.03 MB PDF) [file pcbi.1000990.s002.pdf]

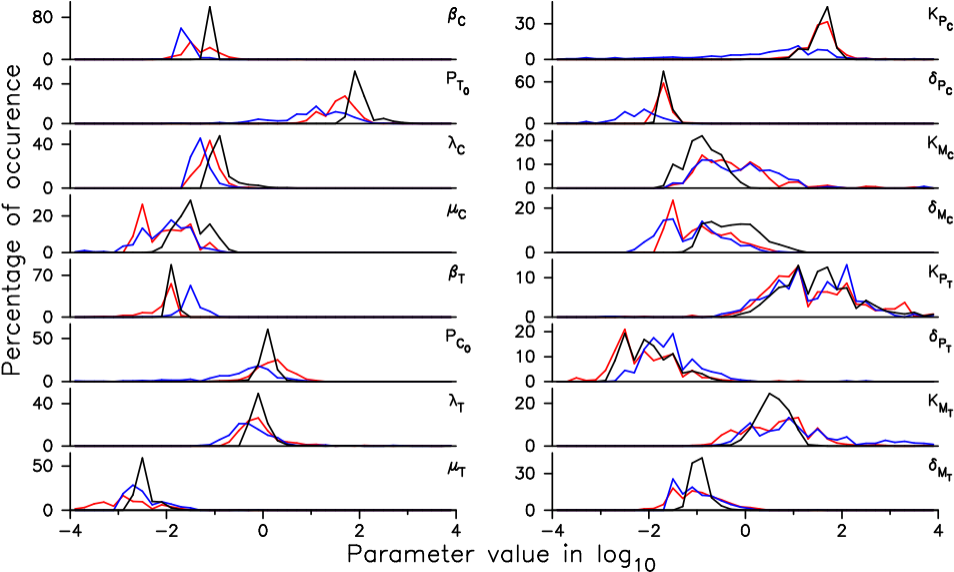

Supplement: Figure S3 — Probability distribution for parameter values in parameter sets with adjustment RMS error below 10%. Parameters are determined as explained in Methods. The percentage of occurrence is evaluated for bins of width 0.2 in log10. The probability distributions of parameter values for the model with all parameters modulated are shown in red and blue for the day and night values, respectively. The probability distribution of parameter values for the model with all parameters constant is shown in black. (0.02 MB PDF) [file pcbi.1000990.s003.pdf]

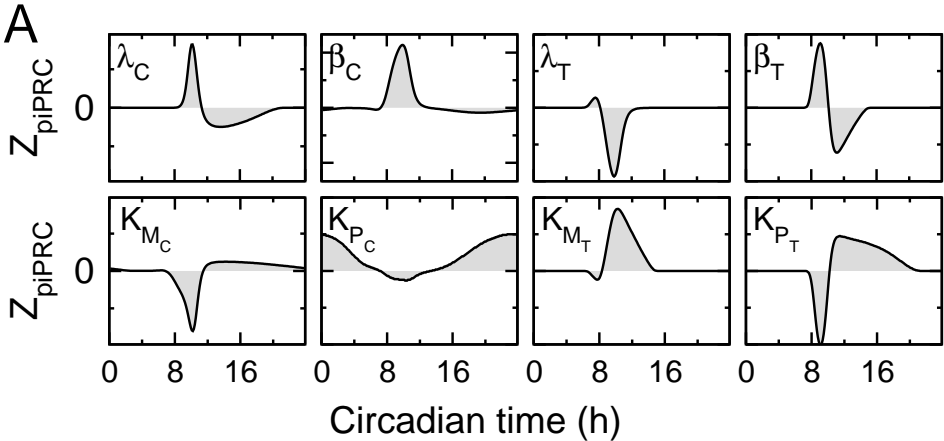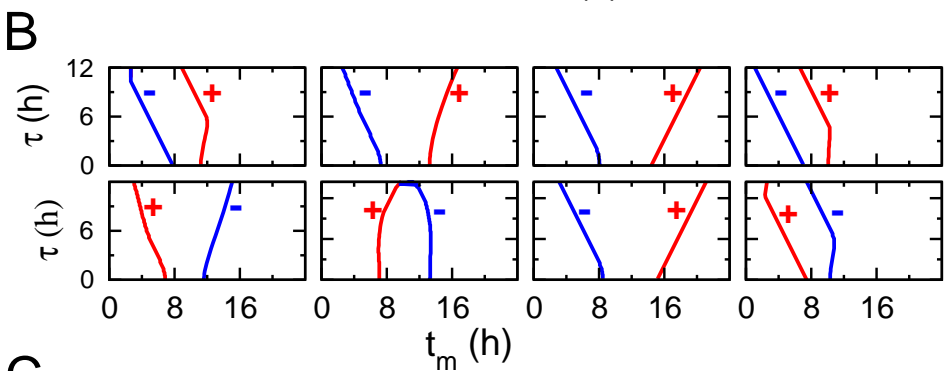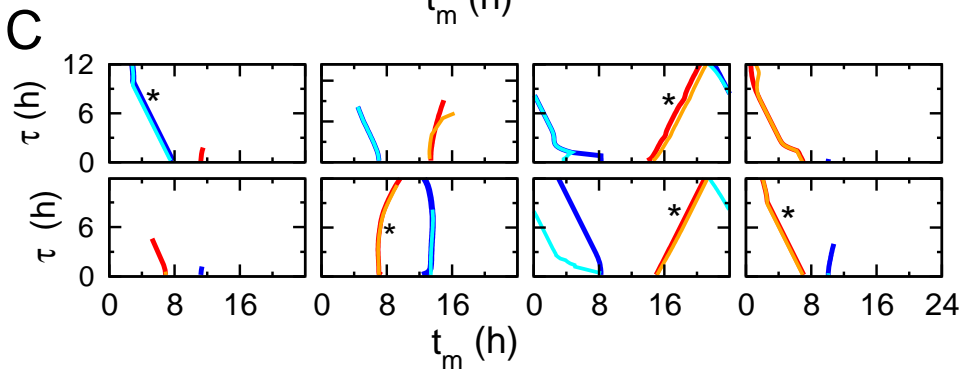

Supplement: Figure S4 — Characterization of coupling schemes. (A) iPRC characterizing the phase change induced by an infinitesimal perturbation of parameters λX, βX and KX. (B) Characterization of time position, tm, and duration τ of couplings with a rectangular gating profile satisfying Eq. (1). Parameters are modulated either positively (red) or negatively (blue). (C) Characterization of time position and duration of couplings with a rectangular gating profile adjusting experimental data with a RMS error below $10% for four different levels of coupling strength (blue: p/p0 = 1.17; cyan: p/p0 = 2; red: p/p0 = 0.85; orange: p/p0 = 0.5; p/p0 being the ratio between the parameter values within and outside the coupling window. (0.03 MB PDF) [file pcbi.1000990.s004.pdf]

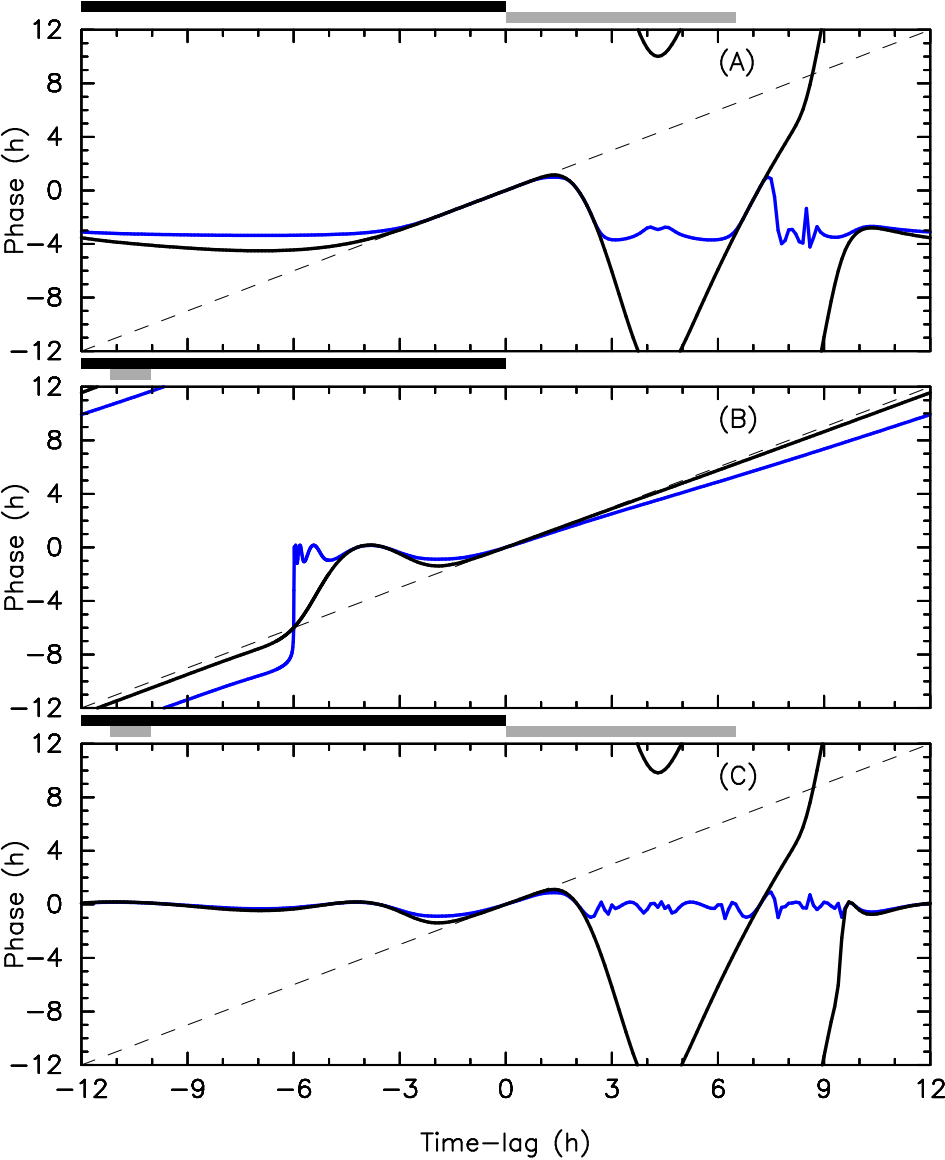

Supplement: Figure S5 — Resetting of the clock model of Fig. 4 in response to a phase shift of the day/night cycle. Solid curves display the residual phase shift of the clock after 1 (black) and 5 (blue) day/night cycles as a function of the initial phase shift. (A) TOC1 degradation rate is multiplied by 2.1 between ZT0 and ZT6.5. (B) CCA1 degradation rate is multiplied by 0.6 between ZT12.8 and ZT13.95. (C) Figure 6C is reproduced here for convenience. TOC1 (resp. CCA1) is multiplied by 2.1 (resp. 0.6) between ZT0 and ZT6.5 (resp. ZT12.8 and ZT13.95), which results in uniform convergence to phase-locking. Phase RMS error after 5 day/night cycles is 25∼min while the maximum error is 1∼hour. (0.03 MB PDF) [file pcbi.1000990.s005.pdf]

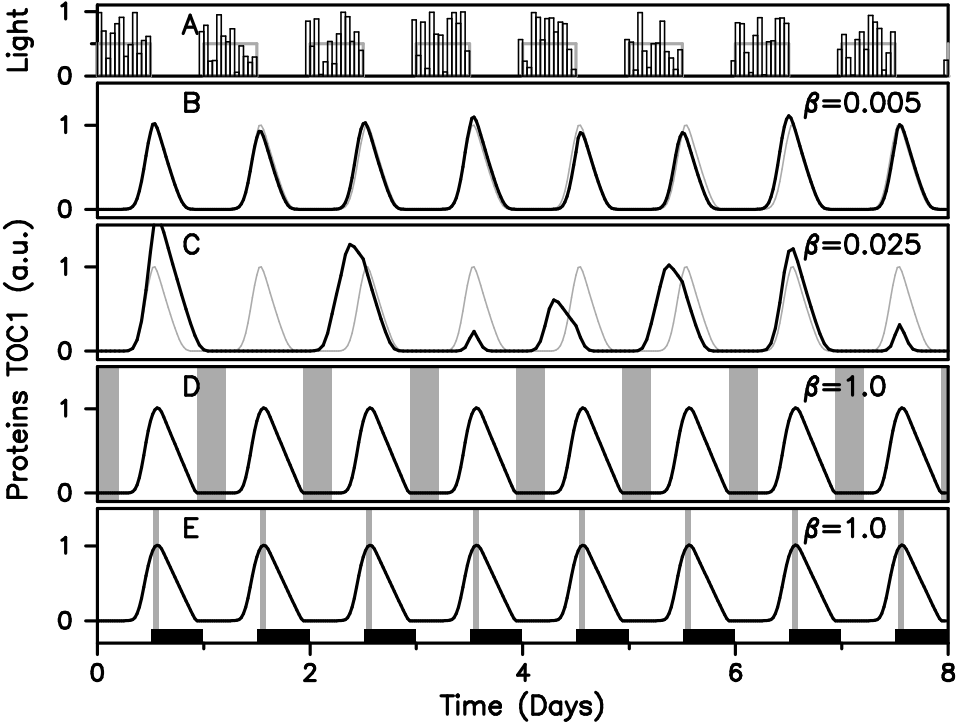

Supplement: Figure S6 — Response of the fully coupled and occasionally coupled clock models to fluctuations in daylight intensity occurring on a time scale of one hour. The figure is otherwise similar to Fig 8. (0.03 MB PDF) [file pcbi.1000990.s006.pdf]

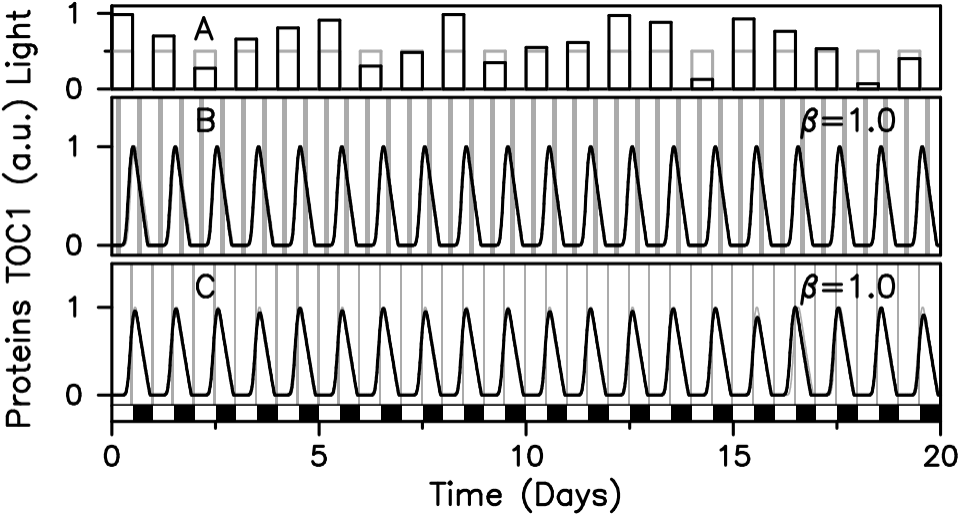

Supplement: Figure S7 — Response of the two occasionally coupled clock models of Fig.∼8 to fluctuations in daylight intensity. (a) Light intensity varying randomly from day to day. The time evolution of TOC1 protein concentration is shown for: (b) the clock model with a FRP of 23.5h; (c) the clock model with a FRP of 25h. The figure is otherwise similar to Fig∼8. (0.06 MB PDF) [file pcbi.1000990.s007.pdf]
