# Supplementary material for: Robustness of Circadian Clocks to Daylight Fluctuations: Hints from the Picoeucaryote Ostreococcus tauri
Source: PLoS Comput Biol. 2010 Nov 11;6(11):e1000990. doi: 10.1371/journal.pcbi.1000990 (PMC2978692; doi:10.1371/journal.pcbi.1000990)
Supplement: Text S1 — Characterization of gated coupling mechanisms in the weak modulation limit. (0.07 MB PDF) [file pcbi.1000990.s008.pdf]

Characterization of Gated Coupling Mechanisms in the Weak Modulation Limit

Supplement to "Robustness of circadian clocks to daylight fluctuations: hints from the picoeucaryote Ostreococcus tauri",
Q. Thommen, B. Pfeuty, P.-E. Morant, F. Corellou, F.-Y. Bouget and M. Lefranc

October 3, 2010

The free oscillator model was shown to adjust remarkably well the RNAmicroarray data from LD12:12 experiments. A tempting hypothesis is that the synchronization of the free running oscillator to the day-night cycle involves a light-dependent gated-coupling mechanism that has restricted effect on the RNA traces when phase locked. We develop here a systematic method to repertoriate the coupling schemes that synchronize the free oscillator to the diurnal cycle while preserving the adjustment score obtained in the absence of coupling. For enough weak coupling strength, any coupling schemes that achieve the correct phase shift preserve the adjustement score. Those coupling schemes can be found in the framework of perturbation theory in the vicinity of a periodic orbit [1,2,3], assuming that the driving force period is enough close to the internal clock period. We consider the state vector of a nonlinear oscillator, which represents the concentration of the molecular clock components. In constant dark conditions, the concentration vector $\mathbf{X}$ evolves according to:

$$d\mathbf{X}/dt = \mathbf{F}(\mathbf{X}, \mathbf{p_0}) \tag{1}$$

Eq. 1 has a periodic solution $\mathbf{X}_\gamma(t)$ corresponding to a stable limit cycle of period T close to 24 hours. We assume that the coupling between the light and the circadian oscillator is mediated by a set of N components (k is the index), which modulate the parameter vector in the direction of $\mathbf{dp}_k$:

$$\mathbf{p}(t) = \mathbf{p_0} + \sum_{k=1,N} L_k(t, \tau_k, (t_m)_k)\mathbf{dp}_k \tag{2}$$

where the $24h$-periodic scalar function $L_k(t, \tau_k, (t_m)_k)$ represents the temporal profile of activation (rectangular- or gaussian-shaped profiles in the present paper) of the light-dependent component k with τ_k and $(t_m)_k$ characterizing the effective coupling window duration and center ($t = 0$ correspond to the night-day transition or CT0).

A small enough parametric impulse perturbation applied at phase u induces an infinitesimal change of the circadian oscillator phase defining a T-periodic scalar function $Z_{piPRC}(u, \mathbf{dp})$ commonly called an infinitesimal phase response curve [2] or a parametric impulse phase response curve [3]:

$$Z_{piPRC}(u, \mathbf{dp}_k) = (\mathbf{dp}_k)^T . \mathbf{Z_p}(u) \tag{3}$$

where

$$\mathbf{Z_p}(u) = \left[\frac{\partial \mathbf{F}(\mathbf{X}_\gamma(u))}{\partial \mathbf{p}}\right]^T \frac{\partial \phi(\mathbf{X}_\gamma(u))}{\partial \mathbf{X}} \tag{4}$$

Then, the phase change induced by the light sensed during the daytime can be derived from the convolution of the temporal profile of the light-sensing components with the piPRC:

$$\Delta\phi = \sum_{k=1,N} \int_0^T L_k(u, \tau_k, (t_m)_k) Z_{piPRC}(u + \phi, \mathbf{dp}_k) du \tag{5}$$

where ϕ is the phase of the oscillator at CT0. A stable entrainment state requires that the scalar functions L and Z_{ipPRC} satisfy:

$$\begin{cases} \sum_{k=1,N} \int_0^T L_k(u, \tau_k, (t_m)_k)\, Z_{piPRC}(u + \phi^*, \mathbf{dp}_k) du = \delta\phi^* \\ \sum_{k=1,N} \int_0^T L_k(u, \tau_k, (t_m)_k)\, Z'_{piPRC}(u + \phi^*, \mathbf{dp}_k) du < 0 \end{cases} \tag{6}$$

where ϕ^* is the locked phase (relative to CT0) and $\delta\phi^*$ is the phase change induced by the period mismatch between the free oscillator and the day-night period, which is assumed to be small with respect to T. The derivative of the function Z_{ipPRC} with respect to the phase variable is noted Z'_{ipPRC}.

For any modulated parameter set **dp** whose Z_{piPRC}-function is equal to $\delta\phi^*$, one can always find τ_k and $(t_m)_k$, that satisfies Eqs. 6 above. In the case where there is a unique coupling scheme ($N = 1$) with a rectangular profile, the coupling interval satisfies:

$$\begin{cases} \int_{t_m-\tau/2}^{t_m+\tau/2} Z_{piPRC}(u, \mathbf{dp}) du = \delta\phi^* \\ \int_{t_m-\tau/2}^{t_m+\tau/2} Z'_{piPRC}(u, \mathbf{dp}) du < 0 \end{cases} \tag{7}$$

Figures 5 and S4 show the numerical solutions of this equation with $\delta\phi^*$ equal to 0 (the FRP being equal to 24 hours), which determine the coupling intervals (compatible with experimental data) for positive and negative modulation of the 16 parameters of the model.

References

[1] Kramer MA, Rabitz H, Calo JM (1984) Sensitivity analysis of oscillatory systems. Appl Math Model. 8: 328-340.

[2] Rand DA, Shulgin BV, Salazar D, Millar AJ (2004) Design principles underlying circadian clocks. J R Soc Interface. 1: 119-30.

[3] Taylor SR, Gunawan R, Petzold LR, Doyle FJ 3rd (2008) Sensitivity Measures for Oscillating Systems: Application to Mammalian Circadian Gene Network. IEEE Trans Automat Contr. 53: 177-188.
